# Co-Expression of an IL-15 Superagonist Facilitates Self-Enrichment of GD_2_-Targeted CAR-NK Cells and Mediates Potent Cell Killing in the Absence of IL-2

**DOI:** 10.3390/cancers15174310

**Published:** 2023-08-29

**Authors:** Malena Bodden, Aline Häcker, Jasmin Röder, Anne Kiefer, Congcong Zhang, Anita Bhatti, Jordi Pfeifer Serrahima, Evelyn Ullrich, Ines Kühnel, Winfried S. Wels

**Affiliations:** 1 Georg-Speyer-Haus, Institute for Tumor Biology and Experimental Therapy, 60596 Frankfurt, Germany; 2 Frankfurt Cancer Institute, Goethe University, 60590 Frankfurt, Germany; 3 German Cancer Consortium (DKTK), Partner Site Frankfurt/Mainz, a Partnership between DKFZ and University Hospital Frankfurt, 60590 Frankfurt, Germany; 4 Department of Pediatrics, Experimental Immunology and Cell Therapy, Goethe University, 60590 Frankfurt, Germany

**Keywords:** natural killer cells, NK-92, chimeric antigen receptor, GD_2_, interleukin-15

## Abstract

**Simple Summary:**

The disialoganglioside GD_2_ is produced at high levels by neuroblastomas and other tumors of neuroectodermal origin, where its expression correlates with increased tumor progression and poor prognosis. Although established therapies with GD_2_-specific antibodies can be efficacious, more effective treatment options are needed for advanced and relapsed tumors. The aim of this work was the generation and functional evaluation of natural killer cells engineered with a GD_2_-specific chimeric antigen receptor (CAR) as a novel off-the-shelf adoptive immunotherapy approach. In preclinical in vitro models, these CAR-NK cells displayed high and selective cytotoxicity against GD_2_-expressing tumor cells. Moreover, GD_2_-CAR NK cells further modified to express an interleukin (IL)-15 superagonist displayed enhanced functionality also in the absence of exogenous cytokines and modulated proliferation and antitumor activity of surrounding innate immune cells and T lymphocytes.

**Abstract:**

In contrast to T lymphocytes, natural killer (NK) cells do not require prior sensitization but are rapidly activated upon encountering virally infected or neoplastic cells. In addition, NK cells can be safely applied in an allogeneic setting, making them important effector cells for the development of off-the-shelf therapeutics for adoptive cancer immunotherapy. To further enhance their therapeutic potential, here, we engineered continuously expanding NK-92 cells as a clinically relevant model to express a humanized second-generation chimeric antigen receptor (CAR) with a composite CD28-CD3ζ signaling domain (hu14.18.28.z) that targets the disialoganglioside GD_2_, which is expressed at high levels by neuroblastoma cells and other tumors of neuroectodermal origin. In a separate approach, we fused an IL-15 superagonist (RD-IL15) to the GD_2_-CAR via a P2A processing site. Lentivirally transduced NK-92/hu14.18.28.z and NK-92/hu14.18.28.z_RD-IL15 cells both displayed high and stable CAR surface expression and specific cytotoxicity toward GD_2_-positive tumor cells. GD_2_-CAR NK cells carrying the RD-IL15 construct in addition expressed the IL-15 superagonist, resulting in self-enrichment and targeted cell killing in the absence of exogenous IL-2. Furthermore, co-culture with RD-IL15-secreting GD_2_-CAR NK cells markedly enhanced proliferation and cytotoxicity of bystander immune cells in a paracrine manner. Our results demonstrate that GD_2_-CAR NK cells co-expressing the IL-15 superagonist mediate potent direct and indirect antitumor effects, suggesting this strategy as a promising approach for the further development of functionally enhanced cellular therapeutics.

## 1. Introduction

Adoptive immunotherapy with chimeric antigen receptor (CAR)-engineered T cells has demonstrated impressive efficacy in patients with B-cell malignancies and is now regularly applied in standard clinical practice [1]. Approaches to target cancers of solid tumor origin with CAR-engineered lymphocytes have so far proven less effective, owing in part to an immunosuppressive tumor microenvironment, limited persistence of infused effector cells and heterogeneity of target antigen expression within the tumor [2]. Furthermore, while CAR targets for B-cell malignancies are restricted to distinct hematopoietic lineages, most surface antigens of solid tumor cells are overexpressed self-antigens, which can also be found in vital healthy tissues, potentially resulting in severe on-target/off-tumor effects of respective CAR effectors [3].

Of note, a promising safety profile was reported in an early clinical trial in neuroblastoma patients with T cells carrying a first-generation CAR that targets the disialoganglioside GD_2_ [4]. GD_2_ is a sialic acid-bearing glycosphingolipid generated from precursor gangliosides by the GD_2_/GD_3_-synthase, followed by translocation to the plasma membrane and anchoring within lipid rafts. In healthy tissues, GD_2_ is only found at low levels in the central nervous system, on peripheral nerves, dermal melanocytes, and mesenchymal stromal cells [5]. In contrast, GD_2_ is highly expressed in malignancies of neuroectodermal origin, such as neuroblastomas or melanomas, and to a variable extent in other tumor entities, such as sarcomas or cancer stem cells in breast carcinoma [6]. In the first clinical trial with GD_2_-CAR T cells, some of the treated patients experienced a complete response [4,7], encouraging subsequent studies in neuroblastoma and midline glioma patients with GD_2_-specific T cells and invariant NKT cells engineered with more advanced CAR designs [8,9,10,11,12]. In earlier preclinical work, also GD_2_-CAR-modified natural killer (NK) cells were shown to effectively eliminate GD_2_-positive neuroblastoma, melanoma, Ewing sarcoma, and breast carcinoma cells [13,14,15,16].

NK cells are important effectors of the innate immune system and play a decisive role in the early immune response against viral infections and malignant cells. In contrast to T lymphocytes, they do not carry an antigen-specific T-cell receptor and do not require clonal selection and expansion. Instead, they rapidly respond after stimulation of germ-line-encoded activating receptors, such as natural cytotoxicity receptors (NCRs) and NKG2D upon encountering stress-induced ligands on tumor cells. Furthermore, they can be safely applied even in an MHC-unmatched allogeneic setting, making them promising and easily accessible effector cells for the development of adoptive cancer immunotherapies [17,18]. Accordingly, without relying on the varying quality and quantity of a patient’s own immune cells, well-characterized CAR-NK cells can be generated as off-the-shelf therapeutics from different sources, such as the clinically usable cell line NK-92 [19,20,21], healthy donor-derived peripheral blood and umbilical cord blood NK cells [22,23,24], or NK cells derived from genetically modified iPSCs [25].

Here, we employed continuously expanding human NK-92 cells to evaluate the antitumor activity of a fully humanized GD_2_-specific second-generation CAR. Unmodified NK-92 cells as well as CAR-engineered derivatives have previously been tested in early phase clinical trials, demonstrating safety and feasibility of this approach [26,27,28]. Like all NK cells, NK-92 cells require the common γ-chain cytokine interleukin (IL)-2 or related IL-15 to retain viability, proliferative capacity, and antitumor activity [29]. While NK cells do not produce these cytokines endogenously, ectopic expression of IL-15 in CAR-engineered derivatives can circumvent this dependency during in vitro culture and support long-term engraftment upon adoptive transfer in vivo [24,30,31]. To investigate autocrine and paracrine effects of a more potent, functionally enhanced IL-15 superagonist (RD-IL15) encompassing the sushi domain of IL-15Rα and mutated IL-15_N72D_ [32], here, we co-expressed this molecule together with the GD_2_-CAR in NK-92 cells and compared proliferation, natural cytotoxicity, and CAR-mediated cell killing of RD-IL15-expressing and RD-IL15-negative CAR-NK cells, as well as their influence on the activity of co-cultured bystander immune cells.

## 2. Materials and Methods

### 2.1. Cells and Culture Conditions

EL4 T cell lymphoma, HEK293T embryonic kidney, MDA-MB453 breast carcinoma (all ATCC, Manassas, VA), and SK-MEL-23 melanoma cells [33] were propagated in DMEM medium (Gibco, Thermo Fisher Scientific, Darmstadt, Germany), UKF-NB3 neuroblastoma cells [34] in IMDM medium (Gibco) and K562 erythroleukemia cells (ATCC) in RPMI 1640 medium (Gibco). All cell culture media contained as supplements 10% heat-inactivated FBS (Capricorn Scientific, Ebsdorfergrund, Germany), 2 mM L-glutamine, as well as 100 U/mL penicillin and 100 µg/mL streptomycin (all Gibco). NK-92 cells [35] (kindly provided by NantKwest, Inc., Culver City, CA, USA) were grown in X-VIVO 10 medium (Lonza, Cologne, Germany) with 5% heat-inactivated human AB plasma (German Red Cross Blood Donation Service Baden-Württemberg-Hessen, Frankfurt, Germany) and 100 IU/mL IL-2 (Proleukin; Novartis Pharma, Nürnberg, Germany) as supplements. Peripheral blood mononuclear cells (PBMCs) of healthy donors were isolated from buffy coats using a Ficoll–Hypaque density gradient centrifugation. Peripheral blood NK cells (pNK) were derived from buffy coats using the RosetteSep human NK cell enrichment cocktail (STEMCELL Technologies, Cologne, Germany) following the manufacturer’s recommendations. PBMCs and pNK cells were cultivated in X-VIVO 10 medium with 5% heat-inactivated human AB plasma as a supplement. 

### 2.2. Generation of GD_2_-CAR-Expressing NK-92 Cells

The GD_2_-specific CAR hu14.18.28.z sequence is composed of an immunoglobulin heavy-chain signal peptide, an extracellular single-chain fragment variable (scFv) antibody domain derived from humanized monoclonal antibody 14.18 (hu14.18) [36], a Myc-tag, a modified CD8α hinge region [37], and the transmembrane and intracellular domains of CD28 (UniprotKB: P10747, amino acid residues 151–220), connected to the intracellular domain of CD3ζ (UniprotKB: P20963-3, amino acid residues 52–163). The hu14.18.28.z_RD-IL15 sequence in addition contains a fusion of an immunoglobulin heavy-chain signal peptide, the sushi domain of IL-15Rα (UniprotKB: Q13261-1, amino acid residues 31–107), and affinity-optimized IL-15_N72D_ [32] linked to the CAR via a porcine teschovirus self-cleaving peptide (P2A). Codon-optimized sequences encoding hu14.18.28.z and RD-IL15 were de novo synthesized (GeneArt, Thermo Fisher Scientific) and assembled upstream of an IRES and enhanced green fluorescent protein (EGFP) cDNA in lentiviral transfer plasmid pHR’SIN-cPPT-SIEW (pSIEW) [38], yielding the vectors pS-hu14.18.28.z-IEW and pS-hu14.18.28.z_RD-IL15-IEW. VSV-G pseudotyped vector particles were generated using HEK293T cells and employed for transduction of NK-92 cells as previously described [37]. EGFP-expressing NK-92 cells were enriched by flow-cytometric cell sorting with a FACSAria Fusion Flow Cytometer (BD Biosciences, Heidelberg, Germany). CAR expression on the cell surface was determined by flow cytometry with an AF647-conjugated Myc-tag-specific antibody (9E10; Invitrogen, Thermo Fisher Scientific) using a FACSCanto II flow cytometer (BD Biosciences). Total CAR protein levels were analyzed by SDS-PAGE of NK-92 cell lysates (30 μg of total proteins per lane) under reducing conditions, followed by immunoblot analysis with CD8α-specific antibody (H-160; Santa Cruz, Heidelberg, Germany), HRP-conjugated secondary antibody (Sigma-Aldrich, Taufkirchen, Germany), and chemiluminescent detection.

### 2.3. Generation of Recombinant Derivatives of Anti-Idiotypic Antibody Ganglidiomab

The IgG_4_-based ganglidiomab scFv-Fc sequence was derived by in silico assembly of an immunoglobulin heavy chain signal peptide and a scFv fragment of anti-idiotypic antibody ganglidiomab, which selectively binds GD_2_-specific antibodies ch14.18 and hu14.18 [39], followed by hinge, CH2, and CH3 domains of human IgG4. Upon codon optimization and de novo synthesis (GeneArt), the fusion gene was inserted into mammalian expression vector pcDNA3. Following transient transfection of HEK293T cells with the resulting plasmid, the recombinant scFv-Fc antibody was purified from the culture supernatant using affinity chromatography employing a HiTrap Protein-G column on an ÄKTA FPLC system (GE Healthcare Europe, Freiburg, Germany). For expression as a GD_2_ surrogate on the surface of target cells, the ganglidiomab scFv sequence was fused to a Myc-tag, a modified CD8α hinge region, and the transmembrane domain of CD28 upstream of IRES and EGFP sequences in lentiviral transfer plasmid pSIEW, yielding pS-gTM-IEW. VSV-G-pseudotyped vector particles were used for the transduction of GD_2_-negative MDA-MB453 cells, and EGFP-positive cells were enriched by flow cytometry.

### 2.4. Analysis of GD_2_ Expression and Cell Killing Activity

Expression of GD_2_ and ganglidiomab scFv (gTM) as a GD_2_ surrogate on the surface of target cells was examined by flow cytometry using a PE-conjugated GD_2_-specific antibody (BioLegend, Koblenz, Germany). Specific cytotoxicity of CAR-NK cells was analyzed in FACS-based assays. After labeling with calcein violet AM (CV) (CellTrace, Invitrogen), tumor cells were incubated with parental or engineered NK-92 cells at effector to target (E/T) ratios of 10:1, 5:1, and 1:1 for 4 h at 37 °C in the absence or presence of 12.5 nM of recombinant ganglidiomab scFv-Fc as a competitor. After co-incubation, 80 µL of a 1 µg/mL propidium iodide (PI) solution were added, followed by analysis of cells with a FACSCanto II flow cytometer. Target cells incubated in the absence of NK-92 cells served as control for spontaneous lysis. CV and PI double positive cells were identified as dead target cells. Specific cytotoxicity was calculated by subtracting spontaneous target cell lysis. Data were analyzed with FACSDiva software (BD Biosciences, https://www.bdbiosciences.com/ja-jp/products/software/instrument-software/bd-facsdiva-software, accessed on 26 June 2023).

For time-lapse imaging experiments, UKF-NB3 cells stained with Far Red (CellTrace, Invitrogen) and carboxyfluorescein succinimidyl ester (CFSE)-labeled NK-92/hu14.18.28.z_RD-IL15 cells were co-incubated at an E/T ratio of 5:1 for 4 h at 37 °C. For imaging, a CQ1 Confocal Quantitative Image Cytometer (Yokogawa, Tokyo, Japan) was used, with phase-contrast and fluorescent images acquired every 5 min at 20× magnification by sample excitation with lasers at 405, 488, and 640 nm. Images were captured at four z-stacks and processed to maximum intensity projections (MIP). Caspase-3 activation was visualized with fluorescent NucView 405 caspase substrate (Sigma-Aldrich).

### 2.5. Analysis of RD-IL15 Expression and Activity

Secretion of RD-IL15 was analyzed by sandwich ELISA using the supernatant of NK-92/hu14.18.28.z_RD-IL15 cells cultured for 3 days at a cell density of 1 × 10^6^ cells/mL in growth medium lacking IL-2, an IL-15-specific capture antibody (34593; R&D Systems, Wiesbaden, Germany), a biotin-conjugated IL-15Rα-specific detection antibody (R&D Systems), and HRP-coupled streptavidin (Genscript, Piscataway, NJ, USA). Recombinant His-tagged RD-IL15 served as a standard. Culture supernatants of parental NK-92 and NK-92/hu14.18.28.z cells were included as controls. RD-IL15 bound to the surface of NK-92/hu14.18.28.z_RD-IL15 cells was detected by flow cytometry with an IL-15-specific antibody (34559; R&D Systems) followed by APC-coupled secondary antibody using a FACSCanto II flow cytometer.

The proliferation of NK-92/hu14.18.28.z_RD-IL15 cells was tested by growing the cells for 7 days in media with or without IL-2. The number of viable cells was determined on days 0, 1, 4, and 7 by trypan blue exclusion. Parental NK-92 and NK-92/hu14.18.28.z were included for comparison. The potential self-enrichment of RD-IL15-expressing CAR-NK cells was analyzed by determining the proportion of EGFP-positive cells over time by flow cytometry in an unsorted population of NK-92 cells freshly transduced with S-hu14.18.28.z_RD-IL15-IEW lentiviral vector and then transferred to growth medium lacking IL-2. To assess the activation of STAT5 as a downstream effector of IL-15 receptor signaling, NK-92/hu14.18.28.z_RD-IL15 cells were starved overnight in IL-2-free growth medium. Cell lysates were prepared and used for SDS-PAGE (30 μg of total proteins per lane) and immunoblot analysis with STAT5-specific (89/Stat5; BD Biosciences) and phospho-STAT5-specific antibodies (C11C5; Cell Signaling Technology, Leiden, The Netherlands) followed by HRP-conjugated secondary antibody (Sigma-Aldrich) and chemiluminescent detection. For comparison, lysates of parental NK-92 cells cultured with or without IL-2 and NK-92/hu14.18.28.z_RD-IL15 cells kept in IL-2 containing medium were included.

### 2.6. Surface Marker Analysis

NK-92, NK-92/hu14.18.28.z, and NK-92/hu14.18.28.z_RD-IL15 cells were incubated at 37 °C with K562 and EL4 target cells at an initial E/T ratio of 1:2, with fresh target cells again added to the effector cells after 24, 48, and 72 h. One day later, surface expression of marker proteins was analyzed by flow cytometry with fluorochrome-conjugated antibodies specific for NKp30 (p30-15; BD Biosciences), NKp44 (p44-8; BD Biosciences), NKp46 (9E2; Miltenyi Biotec, Bergisch Gladbach, Germany), NKG2D (BAT221; Miltenyi Biotec), NKG2A (REA110; Miltenyi Biotec), KIR2D (NKVFS1; Miltenyi Biotec), CD96 (NK92.39; Invitrogen), PD-1 (EH12.2H7; BioLegend), PD-L1 (MIH1; BD Biosciences), LAG-3 (7H2C65; BioLegend), TIGIT (MBSA43; Invitrogen), and TIM-3 (F38-2E2; BioLegend) using a FACSCanto II flow cytometer. Parental and CAR-engineered NK-92 cells cultured without target cells served as controls. Data were analyzed using FACSDiva software.

### 2.7. Transwell Assays

Paracrine stimulation of bystander immune cells by RD-IL15-secreting CAR-NK cells was analyzed in co-culture experiments using transwell inserts (Greiner Bio-One, Frickenhausen, Germany) that allow diffusion of soluble factors. To investigate the effect of RD-IL15 on antitumor activity, PBMCs from healthy donors were cultured with NK-92/hu14.18.28.z_RD-IL15 cells for 16 h at 37 °C in IL-2-free growth medium, followed by direct co-incubation of the pre-stimulated PBMCs with K562 cells at an E/T ratio of 20:1 for 2 h. PBMCs incubated in growth medium without stimulation, in medium supplemented with recombinant human IL-15, or co-cultured with CAR-NK cells lacking RD-IL15 expression served as controls. Cell killing activity was determined as described above for CAR-NK cells.

The effect of RD-IL15 on the proliferation of peripheral blood NK cells from healthy donors was tested by co-incubating CFSE-labeled pNK cells with NK-92/hu14.18.28.z_RD-IL15 cells in transwell assays for 8 days at 37 °C in growth medium lacking IL-2, thereby replacing NK-92/hu14.18.28.z_RD-IL15 cells with fresh cells and medium at day 4. pNK cells stimulated with recombinant IL-15 or co-cultured with CAR-NK cells lacking RD-IL15 expression were included as controls. Proliferating pNK cells were identified using a FACSCanto II flow cytometer according to their decreasing CFSE signal. Using the same basic setup and controls as for pNK cells, proliferation of CD8-positive T cells co-cultured in transwells with NK-92/hu14.18.28.z_RD-IL15 cells was analyzed in mixed lymphocyte reactions, whereby irradiated CD3-negative PBMCs from one donor served as stimulator cells for CFSE-labeled PBMCs from a second donor. Proliferating CD8-positive T cells were then identified according to their decreasing CFSE signal. Gating of CD3^−^ CD56^+^ pNK and CD3^+^ CD8^+^ T cells was performed using CD56- (NCAM16.2; BD Biosciences), CD3- (UCHT1; BioLegend), and CD8-specific antibodies (RPA-T8; BD Biosciences).

### 2.8. Statistical Analysis

Quantitative data are presented as mean with standard deviation (SD). Statistical significance was determined using Welch’s unequal variances *t*-test. All analyses were performed using Prism 9.5.1 software (GraphPad Software, San Diego, CA, USA). 

## 3. Results

### 3.1. Generation of GD_2_-Specific CAR-NK Cells

**Figure 1:**
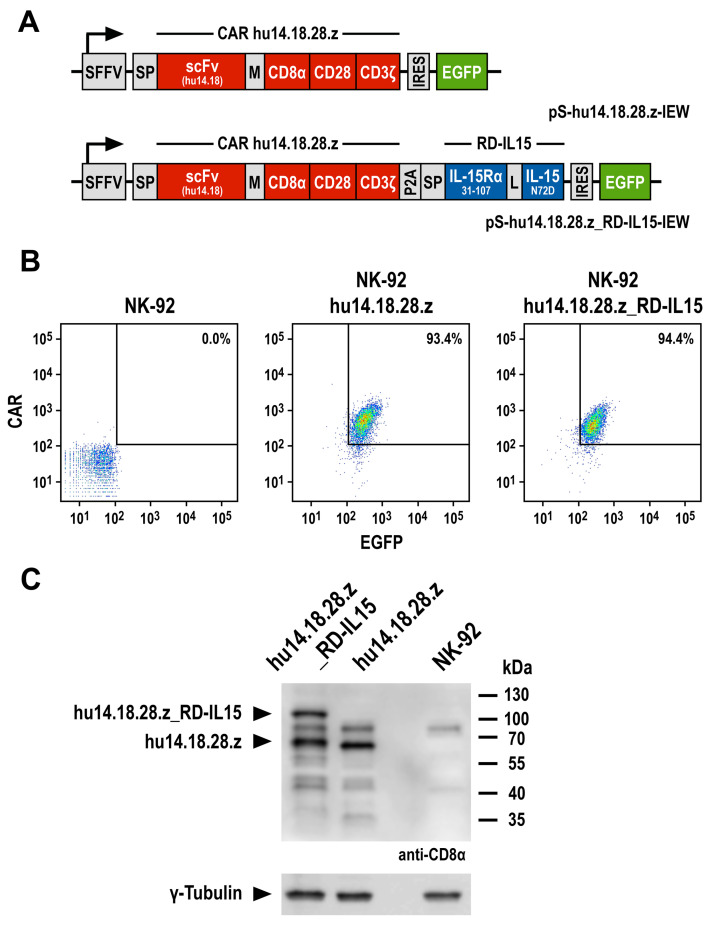
Generation of GD_2_-specific CAR-NK cells: (**A**) Lentiviral transfer plasmids encoding GD_2_-specific chimeric antigen receptor hu14.18.28.z under the control of the Spleen Focus Forming Virus promoter (SFFV). The CAR consists of an immunoglobulin heavy chain signal peptide (SP), a single chain fragment variable (scFv) antibody domain derived from humanized GD_2_-specific antibody 14.18, a Myc-tag (M), a CD8α hinge region (CD8α), transmembrane and intracellular domains of CD28, and the intracellular domain of CD3ζ. For co-expression of the IL-15 superagonist RD-IL15, a sequence encompassing a second signal peptide (SP), the IL-15Rα sushi domain (IL-15Rα_31-107_), a peptide linker (L), and mutated IL-15 (IL-15_N72D_) was fused in frame to the CAR via a porcine teschovirus self-cleaving peptide (P2A). The transgenes are followed by an internal ribosome entry site (IRES) and enhanced green fluorescent protein (EGFP) cDNA. (**B**) Flow cytometric analysis of sorted NK-92/hu14.18.28.z and NK-92/hu14.18.28.z_RD-IL15 cells according to their EGFP and CAR expression. CAR hu14.18.28.z was detected on the cell surface with Myc-tag-specific antibody. Parental NK-92 cells served as control. (**C**) Lysates of CAR-engineered and parental NK-92 cells were subjected to SDS-PAGE under reducing conditions and subsequent immunoblotting with CD8α-specific antibody. The positions of CAR hu14.18.28.z and the remaining amounts of unprocessed hu14.18.28.z_RD-IL15 fusion protein are indicated by arrowheads. γ-Tubulin served as loading control. Original blots can be found in Figure S5.

To target NK cells to cancer cells that express the disialoganglioside GD_2_ as a tumor-associated surface antigen, we generated a codon-optimized and fully humanized chimeric antigen receptor (CAR hu14.18.28.z), which harbors an immunoglobulin heavy-chain signal peptide and a single-chain fragment variable (scFv) domain derived from humanized GD_2_-specific antibody hu14.18 [36], linked to intracellular CD28 and CD3ζ signaling domains via a Myc-tag, an optimized CD8α hinge region, and the CD28 transmembrane domain. In a second approach, the coding sequence of an IL-15 superagonist (RD-IL15) encompassing a signal peptide, the sushi domain of IL-15Rα, and affinity-enhanced IL-15_N72D_ was directly fused to the CAR via a porcine teschovirus P2A self-cleaving peptide. The CAR and CAR/RD-IL15 sequences were inserted into the self-inactivating lentiviral vector pSIEW, with their expression under control of the Spleen Focus Forming Virus (SFFV) promoter linked to that of an EGFP marker gene (Figure 1A). VSV-G pseudotyped lentiviral vector particles were produced and employed to transduce human NK-92 cells, a clinically relevant cell line previously used for the generation of clinical-grade off-the-shelf CAR-NK cell therapeutics [20,28]. Homogeneous cell populations of EGFP-positive NK-92/hu14.18.28.z and NK-92/hu14.18.28.z_RD-IL15 cells were derived by flow cytometric cell sorting, and CAR surface expression by these cells was confirmed by flow cytometry using an antibody specific for the Myc-tag included in the extracellular domain of the CAR sequence. Thereby, no difference in CAR expression levels was observed between NK-92/hu14.18.28.z and NK-92/hu14.18.28.z_RD-IL15 cells (Figure 1B). Also, in immunoblot analysis of whole cell lysates, the CAR hu14.18.28.z protein was detected at similar levels in NK-92/hu14.18.28.z and NK-92/hu14.18.28.z_RD-IL15 cells. Of note, in lysates of RD-IL15-expressing cells, a second specific band was observed, which represents the remaining unprocessed hu14.18.28.z_RD-IL15 fusion protein due to incomplete P2A cleavage of the precursor molecule (Figure 1C and Supplementary Figure S5).

### 3.2. Cell Killing Activity of GD_2_-CAR-Engineered NK-92 Cells

**Figure 2:**
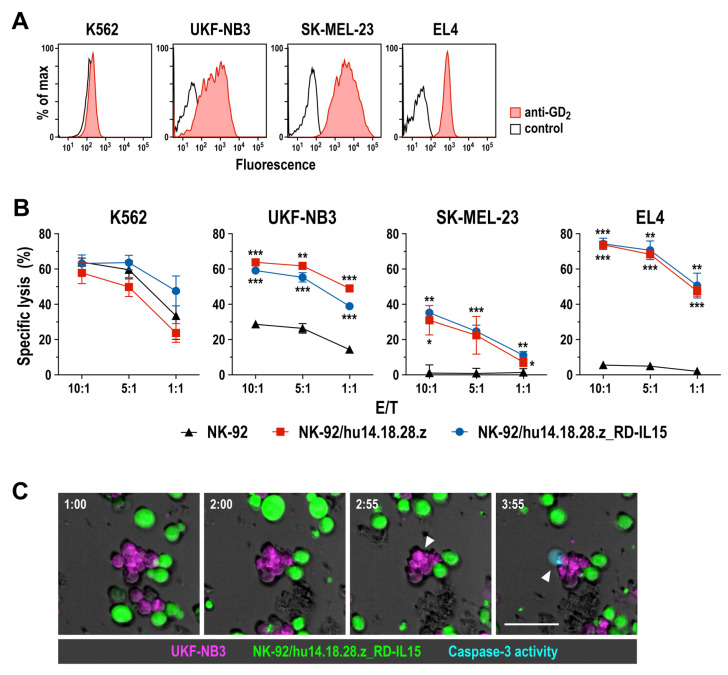
Targeted cytotoxicity of CAR-NK cells against GD_2_-positive tumor cells: (**A**) Surface expression of GD_2_ on established tumor cell lines was investigated by flow cytometry with a GD_2_-specific antibody (filled areas). Unstained cells served as control (black lines). (**B**) Cell killing activity of NK-92/hu14.18.28.z and NK-92/hu14.18.28.z_RD-IL15 cells against GD_2_-negative K562 erythroleukemia cells, and GD_2_-positive UKF-NB3 neuroblastoma, SK-MEL-23 melanoma, and EL4 T cell lymphoma cells was investigated in flow cytometry-based cytotoxicity assays at the indicated effector to target (E/T) ratios after 4 h of co-culture. Parental NK-92 cells were included for comparison. Mean values ± SD are shown; n = 3 independent experiments. Star symbols indicate the statistical significance of differences between CAR-engineered and parental NK-92 cells. ***, *p* < 0.001; **, *p* < 0.01; *, *p* < 0.05. (**C**) The interaction of CFSE-labeled NK-92/hu14.18.28.z_RD-IL15 cells (green) with CellTrace Far Red-stained UKF-NB3 neuroblastoma cells (magenta) was analyzed by time-lapse microscopy. Phase-contrast and fluorescent images were taken every 5 min at 20× magnification. Shown are merged images of a representative field taken at the indicated time points (hours:minutes). Formation of apoptotic bodies and membrane blebbing indicate target cell lysis (white arrowheads). Caspase-3 activation was visualized with fluorescent NucView 405 caspase substrate (cyan). E/T ratio: 5:1; scale bar: 50 μm.

To evaluate specific antitumor activity of NK-92/hu14.18.28.z and NK-92/hu14.18.28.z_RD-IL15 cells, we employed established cancer cell lines with different GD_2_ expression levels in in vitro cell killing experiments. These included MHC I-negative human K562 erythroleukemia cells, which do not express GD_2_ but allow assessment of CAR-independent natural cytotoxicity of NK cells, and GD_2_-positive human UKF-NB3 neuroblastoma and SK-MEL-23 melanoma cells, as well as murine EL4 T cell lymphoma cells (Figure 2A). After 4 h of co-incubation at different effector to target (E/T) ratios, NK-92/hu14.18.28.z and NK-92/hu14.18.28.z_RD-IL15 cells displayed similar cytotoxicity against GD_2_-negative K562 cells as parental NK-92, indicating that CAR expression did not affect natural cytotoxicity of the effector cells. In sharp contrast, GD_2_-expressing SK-MEL-23 and EL4 proved highly resistant toward unmodified NK-92, while they were readily killed in a dose-dependent manner and to the same degree by CAR-engineered NK-92/hu14.18.28.z and NK-92/hu14.18.28.z_RD-IL15 cells (Figure 2B). UKF-NB3 cells displayed moderate sensitivity to parental NK-92 cells, which was markedly enhanced if the effector cells expressed the GD_2_-specific CAR. Thereby, the more pronounced sensitivity of UKF-NB3 toward NK-92/hu14.18.28.z and NK-92/hu14.18.28.z_RD-IL15 cells than SK-MEL-23 despite their lower GD_2_ expression suggests additive effects of CAR-mediated cytotoxicity and cell killing triggered by endogenously expressed activating receptors of NK-92. To assess the kinetics of cell killing, we also conducted a time-lapse microscopy experiment with UKF-NB3 and NK-92/hu14.18.28.z_RD-IL15 cells, demonstrating the induction of apoptosis in the target cells within approximately 2 h after initial contact with the CAR-NK cells (Figure 2C).

**Figure 3:**
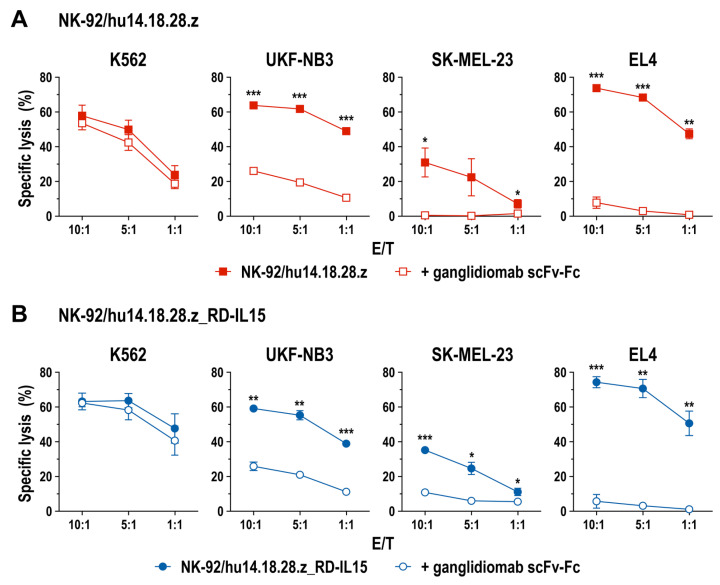
Specificity of CAR-mediated cell killing: CAR-mediated recognition of target cells by NK-92/hu14.18.28.z (**A**) and NK-92/hu14.18.28.z_RD-IL15 cells (**B**) was analyzed in cytotoxicity assays after 4 h of co-incubation at the indicated E/T ratios in the presence or absence of 12.5 nM of ganglidiomab-derived anti-idiotypic scFv-Fc antibody, which blocks the hu14.18 domain of the CAR. Mean values ± SD are shown; n = 3 independent experiments; ***, *p* < 0.001; **, *p* < 0.01; *, *p* < 0.05.

To confirm specificity of CAR-mediated cytotoxicity, we performed quantitative cell killing experiments as described above, including a derivative of anti-idiotypic antibody ganglidiomab as a specific competitor. Ganglidiomab binds selectively to the antigen binding site of 14.18-based anti-GD_2_ antibodies, thereby mimicking GD_2_ and blocking access of the antibodies to their target antigen [39]. For the competition experiments, we generated a recombinant scFv-Fc molecule harboring a scFv antibody fragment of ganglidiomab fused to hinge, CH2, and CH3 domains of human IgG4. This protein displayed specific binding to CAR-expressing NK-92/hu14.18.28.z and NK-92/hu14.18.28.z_RD-IL15 cells, but not to parental NK-92 (Supplementary Figure S1). While the addition of the ganglidiomab scFv-Fc molecule did not affect natural cytotoxicity of the CAR-expressing NK-92 cells against K562, in the presence of the competitor, specific cytotoxicity of NK-92/hu14.18.28.z and NK-92/hu14.18.28.z_RD-IL15 cells against GD_2_-positive targets was either abolished (SK-MEL-23, EL4) or reduced to the level also observed with CAR-negative parental NK-92 cells (UKF-NB3) (Figure 3). In addition to its use as a competitor, we also tested the suitability of recombinant ganglidiomab as a GD_2_ surrogate in cell killing assays with NK-92/hu14.18.28.z and NK-92/hu14.18.28.z_RD-IL15 cells. Indeed, if expressed as a membrane-anchored scFv antibody (termed gTM) on the surface of GD_2_-negative MDA-MB453 breast carcinoma cells, ganglidiomab facilitated specific recognition and killing by the CAR-engineered but not parental NK-92 cells (Supplementary Figure S2).

Taken together, these data demonstrate that CAR hu14.18.28.z is functional and mediates targeted cell killing by the engineered NK-92 cells, without co-expression of the IL-15 superagonist negatively affecting activity of NK-92/hu14.18.28.z_RD-IL15 when compared to NK-92/hu14.18.28.z.

### 3.3. Growth and Functionality of RD-IL15-Expressing CAR-NK Cells in the Absence of IL-2

**Figure 4:**
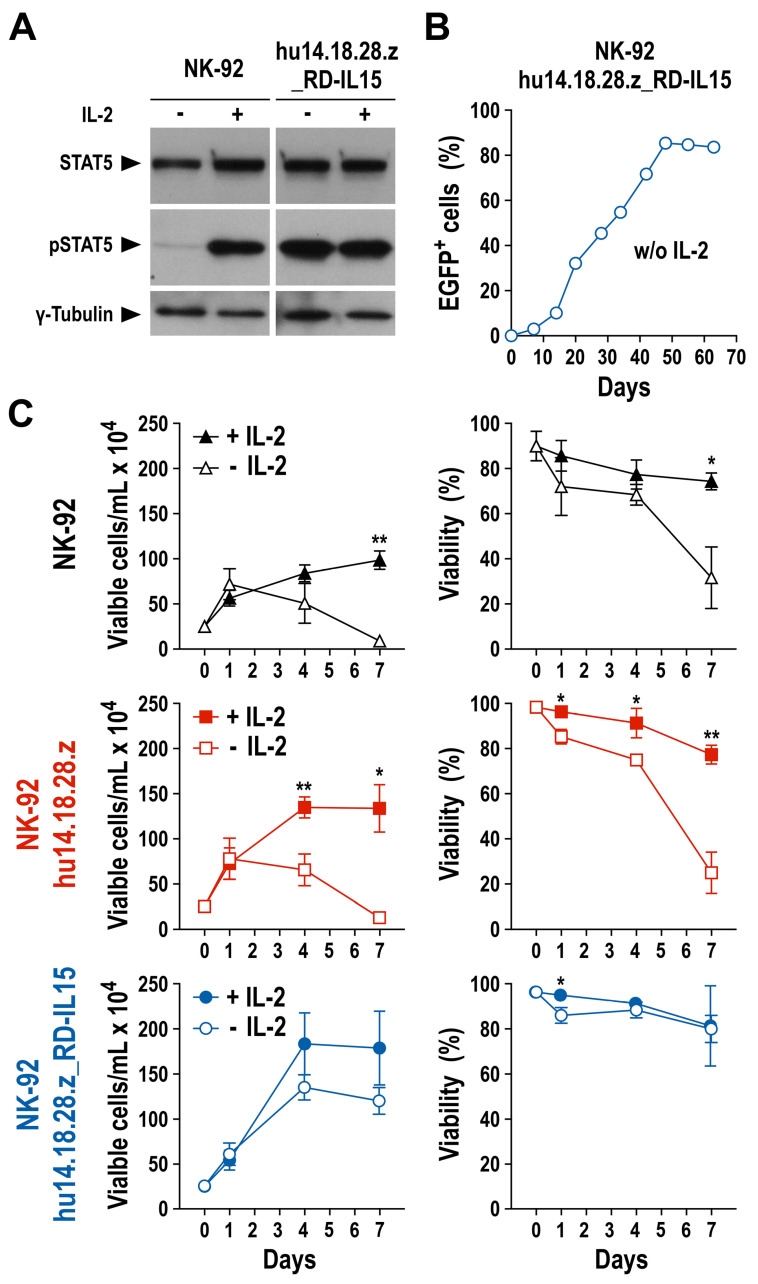
Proliferation of RD-IL15-expressing CAR-NK cells in the absence of IL-2: (**A**) Activation of the IL-15 receptor signaling pathway in NK-92/hu14.18.28.z_RD-IL15 cells. Lysates of NK-92/hu14.18.28.z_RD-IL15 or parental NK-92 cells cultured for 24 h in medium with or without IL-2 were subjected to SDS-PAGE under reducing conditions and subsequent immunoblotting with antibodies specific for total and phosphorylated STAT5. γ-Tubulin served as a loading control. Original blots can be found in Figure S6. (**B**) Self-enrichment of RD-IL15-expressing CAR-NK cells over time was assessed by flow cytometric analysis of the proportion of EGFP-positive cells in cultures of NK-92 cells transduced with lentiviral vector S-hu14.18.28.z_RD-IL15 and then kept in medium lacking IL-2. (**C**) Proliferation and viability of NK-92/hu14.18.28.z and NK-92/hu14.18.28.z_RD-IL15 cells grown for 7 days in the presence (filled symbols) or absence of IL-2 (open symbols). Cell density and proportion of viable cells were determined on days 0, 1, 4, and 7. Mean values ± SD are shown; n = 3; **, *p* < 0.01; *, *p* < 0.05.

To confirm constitutive expression and secretion of the IL-15 superagonist by NK-92/hu14.18.28.z_RD-IL15 cells, we performed FACS experiments that demonstrated the presence of RD-IL15 protein and its binding to the surface of the producer cells (Supplementary Figure S3A). The cytokine could also be detected by sandwich ELISA in the culture supernatant of NK-92/hu14.18.28.z_RD-IL15 cells, but not parental NK-92 and RD-IL15-negative NK-92/hu14.18.28.z cells included as controls (Supplementary Figure S3B). Subsequently, we analyzed potential autocrine stimulation of NK-92/hu14.18.28.z_RD-IL15 cells by the IL-15 superagonist. For this, the cells were kept in the presence or absence of IL-2 for 24 h, before the preparation of whole cell lysates and immunoblot analysis of phosphorylated STAT5 (pSTAT5) as a downstream effector of activated IL-2R/IL-15R complexes. While parental NK-92 cells included as a control only contained activated STAT5 if IL-2 was present, pSTAT5 levels in NK-92/hu14.18.28.z_RD-IL15 cells remained high irrespective of IL-2, indicating that RD-IL15 produced by the cells was biologically active and able to activate IL-2R/IL-15R signaling in an autocrine manner (Figure 4A and Figure S6). Furthermore, when NK-92 cells were freshly transduced with lentiviral S-hu14.18.28.z_RD-IL15-IEW vector and placed in IL-2-free medium immediately after recovery from the transduction, the proportion of EGFP-positive CAR-NK cells increased over time to more than 85% without any cell sorting, indicative of self-enrichment of successfully transduced and RD-IL15-producing cells (Figure 4B). Even if initially propagated in complete medium with IL-2, NK-92/hu14.18.28.z_RD-IL15 cells, but not parental NK-92 or RD-IL15-negative NK-92/hu14.18.28.z cells continued to proliferate and maintained high cell viability upon transfer in IL-2-free medium, demonstrating that the IL-15 superagonist is able to compensate for the lack of IL-2 (Figure 4C).

**Figure 5:**
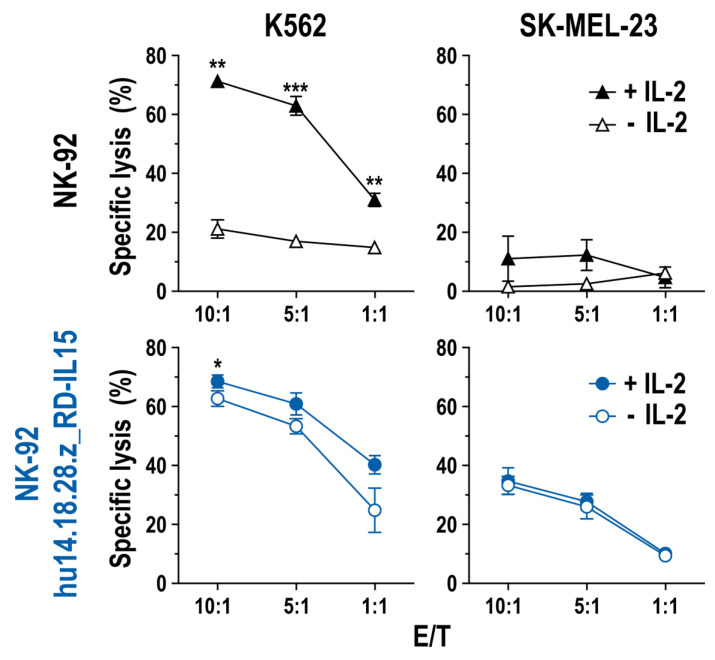
Cell killing activity of RD-IL15-expressing CAR-NK cells in the absence of IL-2: Natural cytotoxicity against GD_2_-negative K562 erythroleukemia and CAR-mediated cell killing against GD_2_-expressing SK-MEL-23 melanoma cells of NK-92/hu14.18.28.z_RD-IL15 cells grown for 3 days in medium lacking IL-2 (open symbols) was investigated in flow cytometry-based cytotoxicity assays at the indicated E/T ratios after co-culture for 4 h. NK-92/hu14.18.28.z_RD-IL15 cells grown in the presence of IL-2 (filled symbols) and parental NK-92 cells were included for comparison. Mean values ± SD are shown; n = 3; ***, *p* < 0.001; **, *p* < 0.01; *, *p* < 0.05.

Next, the impact of RD-IL15 expression on natural and CAR-mediated cytotoxicity of NK-92/hu14.18.28.z_RD-IL15 cells in the absence of IL-2 was analyzed. For this, the RD-IL15-producing CAR-NK cells were cultured for 3 days in IL-2-free medium, before assessing the cytotoxicity against GD_2_-negative but NK-sensitive K562 and GD_2_-positive but otherwise NK-resistant SK-MEL-23 cells. As shown in Figure 5, natural and CAR-mediated cytotoxicity of NK-92/hu14.18.28.z_RD-IL15 cells against K562 and SK-MEL-23 was retained irrespective of the presence of IL-2, while parental NK-92 cells included as a control were inactive against SK-MEL-23 and displayed high cell killing activity against K562 cells only if kept in IL-2-containing medium.

**Figure 6:**
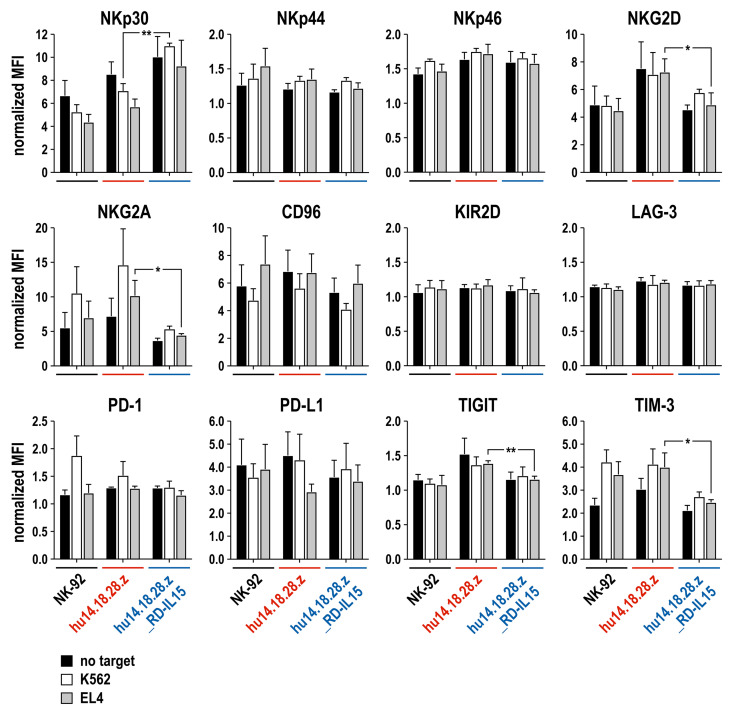
Effect of repeated stimulation on CAR-NK cells: Parental NK-92, NK-92/hu14.18.28.z, and NK-92/hu14.18.28.z_RD-IL15 cells were co-incubated with GD_2_-negative K562 (white bars) or GD_2_-positive EL4 cells (gray bars) at an E/T ratio of 1:2, with fresh target cells added after 24, 48, and 72 h. One day later, cell surface expression of the indicated activating and inhibitory receptors and ligands was analyzed by flow cytometry. Parental and CAR-engineered NK-92 cells cultured without target cells served as controls (black bars). MFI: mean fluorescence intensity, normalized to unstained controls. Mean values ± SD are shown; n = 3; **, *p* < 0.01; *, *p* < 0.05. Representative contour plots are presented in Supplementary Figure S4.

Previous reports indicated that prolonged stimulation of NK cells with IL-15 could adversely affect their effector functions [40,41]. To test whether this may also be the case for NK-92/hu14.18.28.z_RD-IL15 cells, which continuously produce the IL-15 superagonist, we analyzed potential exhaustion of the cells upon repeated exposure to target cells by assessing cell surface expression of a panel of activating and inhibitory receptors and ligands, including the natural cytotoxicity receptors NKp30, NKp44, NKp46, the activating receptor NKG2D, the inhibitory molecules NKG2A, CD96, and KIR2D, the exhaustion markers LAG-3, TIGIT, and TIM-3, as well as the immune checkpoint molecules PD-1 and PD-L1 (Figure 6). RD-IL15-producing CAR-NK cells as well as parental NK-92 and RD-IL15-negative NK-92/hu14.18.28.z cells were kept without target cells, or co-incubated with GD_2_-negative K562 or GD_2_-positive EL4 cells at an E/T ratio of 1:2, with fresh target cells added after 24, 48, and 72 h. Surface marker expression was analyzed on the following day. Thereby, we did not observe an increased expression of any of the tested negative regulators, exhaustion markers, or checkpoint molecules by NK-92/hu14.18.28.z_RD-IL15 cells at steady state or upon repeated activation when compared to NK-92 and NK-92/hu14.18.28.z cells. Instead, we noted a reduced expression of NKG2A and TIM-3 by NK-92/hu14.18.28.z_RD-IL15 cells, both in the absence and presence of target cells, which was most prominent when comparing RD-IL15-expressing and RD-IL15-negative CAR-NK cells after repeated activation with GD_2_-positive EL4 cells. While NK-92/hu14.18.28.z cells with or without target cells displayed an enhanced expression of TIGIT, levels were reduced to values also observed for parental NK-92 cells in the case of NK-92/hu14.18.28.z_RD-IL15.

Overall, the observed changes in the surface expression of the investigated markers between NK-92/hu14.18.28.z_RD-IL15 and NK-92/hu14.18.28.z cells were small and did not result in differences in CAR-independent natural cytotoxicity against GD_2_-negative targets K562 (NK-sensitive; see Figure 2B) and MDA-MB453 (NK-resistant; see Supplementary Figure S2).

Collectively, these results show that NK-92/hu14.18.28.z_RD-IL15 cells secrete biologically active IL-15 superagonist, which stimulates the CAR-NK cells in an autocrine manner without inducing an exhausted phenotype, and allows them to maintain growth as well as high and specific antitumor activity in the absence of exogenous cytokines.

### 3.4. Activation of Bystander Immune Cells by Secreted RD-IL15

**Figure 7:**
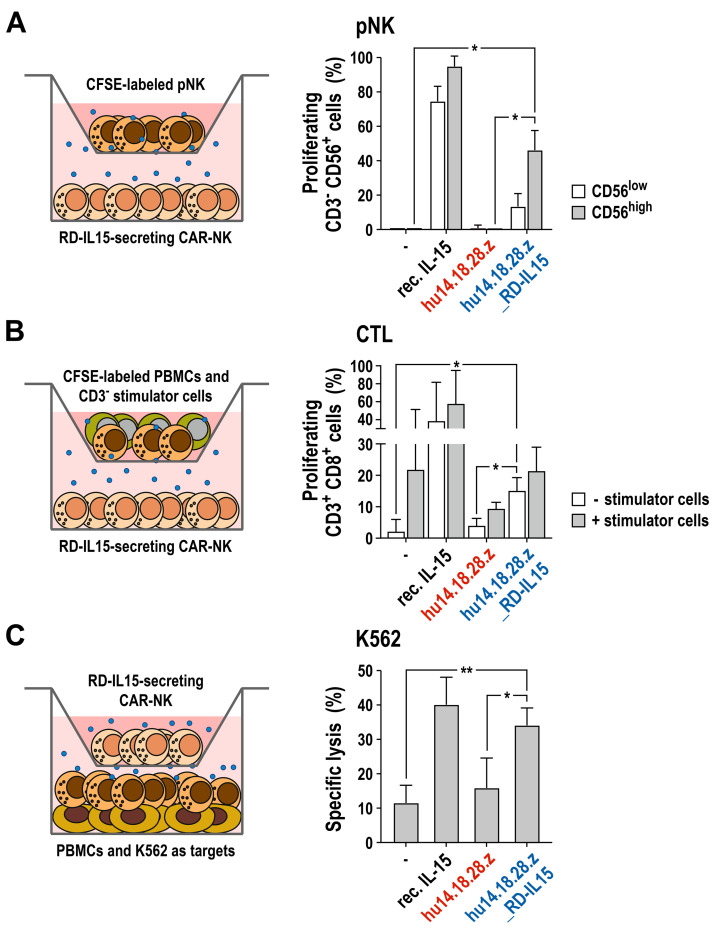
Paracrine stimulation of bystander lymphocytes by secreted RD-IL15: (**A**) Transwell-based proliferation assay with donor-derived primary NK (pNK) cells. CFSE-labeled pNK cells from healthy donors were seeded in transwell inserts and co-cultured with NK-92/hu14.18.28.z_RD-IL15 cells for 7 days in IL-2-free growth medium. The bottom culture of NK-92/hu14.18.28.z_RD-IL15 cells was exchanged with fresh cells and medium on day 4. Proliferating pNK cells were identified by flow cytometry according to their decreasing CFSE signal and subclassified as CD3^−^ CD56^low^ and CD3^−^ CD56^high^ populations using CD3- and CD56-specific antibodies. (**B**) Transwell-based mixed lymphocyte reaction. CFSE-labeled PBMCs from healthy donors were seeded in transwell inserts in the absence or presence of irradiated allogeneic CD3^−^ stimulator cells and co-cultured with NK-92/hu14.18.28.z_RD-IL15 cells for 7 days in IL-2-free medium. The bottom culture of NK-92/hu14.18.28.z_RD-IL15 cells was exchanged with fresh cells and medium on day 4. Proliferating cytotoxic T lymphocytes (CTL) in the cultures were identified by flow cytometry according to their decreasing CFSE signal and staining with CD3- and CD8-specific antibodies. (**C**) Transwell-based cytotoxicity assay. PBMCs from healthy donors were co-cultured for 16 h in IL-2-free growth medium with NK-92/hu14.18.28.z_RD-IL15 cells placed in transwell inserts. Then, K562 cells were added to the pre-stimulated PBMCs at an E/T ratio of 20:1. Cell killing activity was determined after 2 h of co-incubation of PBMCs and K562 cells in a flow cytometry-based cytotoxicity assay. In all experiments, samples co-cultured with NK-92/hu14.18.28.z cells were included for comparison. Primary lymphocytes cultured without CAR-NK cells or stimulated with 20 ng/mL of recombinant IL-15 served as controls. Mean values ± SD are shown; n = 3 different donors; **, *p* < 0.01; *, *p* < 0.05.

To test whether RD-IL15 secreted by NK-92/hu14.18.28.z_RD-IL15 cells can also act in a paracrine manner and support growth and activity of effector lymphocytes in the vicinity of the producer CAR-NK cells, we next performed a set of co-culture experiments with peripheral blood-derived immune cells from three different healthy donors. For this, we used transwell cultures to allow free diffusion of soluble factors produced by the CAR-NK cells but prevent direct cell–cell contacts between primary lymphocytes and NK-92/hu14.18.28.z_RD-IL15 cells. First, we analyzed the proliferation of purified primary NK (pNK) cells in the presence of CAR-NK cells secreting the IL-15 superagonist. pNK cells were labeled with CFSE and then seeded in medium without IL-2 in transwell inserts placed above NK-92/hu14.18.28.z_RD-IL15 cells. Cells were grown for 7 days, with the bottom culture of NK-92/hu14.18.28.z_RD-IL15 cells exchanged with fresh cells and medium on day 4. Proliferating pNK cells were then identified by flow cytometry according to their decreasing CFSE signal (Figure 7A). Thereby, pNK cells stopped proliferation in the absence of IL-2 in control samples without CAR-NK cells or RD-IL15-negative NK-92/hu14.18.28.z cells. In contrast, in the presence of recombinant IL-15 included as a positive control, pNK cells continued to grow in IL-2-free medium. Similarly, transwell co-culture with RD-IL15-secreting CAR-NK cells allowed pNK cells to maintain proliferation in the absence of IL-2, albeit to a lesser extent than recombinant IL-15, likely due to the limited availability of RD-IL15 in the culture supernatant. Thereby, the supporting effect of NK-92/hu14.18.28.z_RD-IL15 cells was more pronounced for pNK cells expressing high levels of CD56, possibly representing typical CD56^bright^ NK cells.

Next, we analyzed the effect of RD-IL15 on CD8-positive T cells by performing a transwell mixed lymphocyte reaction (Figure 7B). As effector cells, CFSE-labeled peripheral blood mononuclear cells (PBMCs) from one donor were seeded in transwell inserts together with irradiated CD3-negative PBMCs from an unrelated second donor as allogeneic stimulator cells. For exposure of the CD8^+^ T cells to soluble factors from the CAR-NK cells, the inserts containing the mixed lymphocyte reactions were then placed above NK-92/hu14.18.28.z_RD-IL15 cells. The transwell cultures were kept in medium without exogenous cytokines for 7 days, with the NK-92/hu14.18.28.z_RD-IL15 cells in the bottom chamber exchanged with fresh cells and medium on day 4. Proliferating CD3^+^ CD8^+^ cytotoxic T cells (CTLs) in the transwell inserts were then identified by flow cytometry according to their decreasing CFSE signal. Recombinant IL-15 added as a positive control to mixed lymphocyte reactions without co-cultured CAR-NK cells stimulated proliferation of CTLs already in the absence of stimulator cells from an unrelated donor and further increased the activating effect of allogeneic stimulator cells. Cultivation of PBMCs without any stimulation did not result in significant proliferation of the CTL subpopulation, which was also the case if co-cultured only with RD-IL15-negative NK-92/hu14.18.28.z cells. In contrast, culture of PBMCs in the absence of allogeneic stimulator cells but in the presence of NK-92/hu14.18.28.z_RD-IL15 cells resulted in a significant increase in proliferating CTLs when compared to co-cultures with NK-92/hu14.18.28.z cells that lack RD-IL15. As expected, the addition of allogeneic stimulator cells to PBMCs enhanced the proliferation of the CTL subpopulation already in the absence of exogenous cytokines or RD-IL15-producing CAR-NK cells, likely supported by IL-2 and other cytokines endogenously produced by the stimulated T cells in the assay. Interestingly, this effect was reduced if NK-92/hu14.18.28.z cells without RD-IL15 were present, possibly due to the partial consumption of the T cell-derived cytokines by the CAR-NK cells. Of note, co-culture with NK-92/hu14.18.28.z_RD-IL15 cells compensated for this negative effect of CAR-NK cells lacking RD-IL15.

Lastly, we investigated the influence of RD-IL15 secreted by the CAR-NK cells on antitumor activity of primary NK cells and other innate effector lymphocytes from peripheral blood. For this, PBMCs from healthy donors were co-cultured for 16 h in IL-2-free medium in the bottom wells, with NK-92/hu14.18.28.z_RD-IL15 cells placed on top in transwell inserts. Then, K562 cells were added to the pre-stimulated PBMCs for 2 h, before specific cytotoxicity of the PBMCs was determined with a flow cytometry-based cell killing assay (Figure 7C). Thereby, co-culture with the RD-IL15-producing CAR-NK cells markedly increased the natural cytotoxicity of PBMCs almost to the same extent as the addition of recombinant IL-15, while co-culture with NK-92/hu14.18.28.z cells lacking RD-IL15 had no significant effect.

Taken together, these data demonstrate that NK-92/hu14.18.28.z_RD-IL15 cells secrete the IL-15 superagonist in amounts sufficient to support the growth of primary NK cells and CTLs in their vicinity and enhance the cytotoxic activity of innate bystander lymphocytes.

## 4. Discussion

High expression of the disialoganglioside GD_2_ is a particular feature of nearly all neuroblastomas but can also be found to a varying degree in other tumors of neuroectodermal origin [6]. For high-risk neuroblastoma, immunotherapy with GD_2_-specific monoclonal antibodies that can trigger antibody-dependent cell-mediated cytotoxicity (ADCC) of NK cells via activation of FcγRIIIa (CD16) has markedly increased survival rates [42]. Nevertheless, remaining limitations of monoclonal antibodies warrant efforts to develop potentially more effective treatment strategies, especially in the case of resistance to antibody therapy despite continued GD_2_ expression by the tumor. In this setting, GD_2_-CAR-engineered lymphocytes may be of particular benefit, since they link antibody-mediated recognition of tumor cells directly with potent intracellular signaling in genetically modified effector lymphocytes, bypassing variable activity of endogenous immune cells and the potential influence of CD16 polymorphisms on antitumor activity [43]. 

In our study, we employed the human NK cell line NK-92 as a clinically relevant model to generate variants that express the fully humanized second-generation CAR hu14.18.28.z, which is based on a scFv domain of GD_2_-specific monoclonal antibody hu14.18, a costimulatory CD28 domain and CD3ζ as a potent trigger of NK-cell effector functions. Although first-generation CARs only containing CD3ζ are active in NK cells [14,19], similar to CAR-T cells, inclusion of costimulatory activity in the CAR can enhance functionality of both primary NK cells and NK-92 [13,22,25,37,44]. In parallel to NK-92/hu14.18.28.z, we generated NK-92/hu14.18.28.z_RD-IL15 cells that carry the same CAR but co-express the IL-15 superagonist RD-IL15 from a bicistronic vector construct. Both CAR-NK cell variants displayed comparable and homogeneous CAR expression on the cell surface. Nevertheless, immunoblot analysis of total cellular proteins indicated incomplete processing of some of the protein expressed from the hu14.18.28.z_RD-IL15 construct. Processing of 2A sequences, such as the P2A site, can be influenced by the preceding amino acid sequence of the fusion partner and may be improved by including an additional GSG linker [45].

NK-92/hu14.18.28.z and NK-92/hu14.18.28.z_RD-IL15 cells exhibited similar natural cytotoxicity as parental NK-92 cells against GD_2_-negative but NK-sensitive K562 cells, and efficiently eliminated GD_2_-positive tumor targets. Confirming CAR-mediated recognition of the cancer cells, in the latter case, cell killing was reduced or abrogated in the presence of a recombinant derivative of anti-idiotypic antibody ganglidiomab, which blocks the interaction of the antigen-binding domain of the hu14.18 scFv fragment with GD_2_ [39]. While SK-MEL-23 melanoma and EL4 T cell lymphoma cells were selectively killed by the CAR-engineered variants but resistant in short-term assays to parental NK-92, in the case of UKF-NB3 neuroblastoma cells, an additive effect of natural and CAR-mediated cytotoxicity was observed, indicated by the reduction in cell killing to the level of unmodified NK-92 cells in the presence of the ganglidiomab-derived competitor. This cooperation of natural and CAR-mediated cytotoxicity could be particularly relevant in the case of inhomogeneous target antigen expression in a tumor and may help to circumvent immune escape by antigen-loss variants. While the functionality of NK-92/hu14.18.28.z and NK-92/hu14.18.28.z_RD-IL15 cells was very similar if kept in the presence of recombinant IL-2, marked differences in the activity of cells expressing the IL-15 superagonist were found if cultured without exogenous cytokines.

Enhancing the functionality of CAR-engineered lymphocytes like T and NK cells by ectopic expression of pro-inflammatory cytokines is an attractive approach to restrict cytokine activity to the tumor vicinity where adoptively transferred effector cells are expected to be localized, thereby reshaping the immunological tumor microenvironment. This could avoid the sometimes detrimental effects of systemic cytokine therapy, in particular if cytokine production is transcriptionally linked to CAR activation [46,47]. In the case of CAR-NK cells and IL-15, constitutive expression of the cytokine transgene independent of CAR stimulation may be preferred due to its ability to also support growth and activity of the producer cells themselves [24,30,48,49]. This circumvents the dependence on exogenous IL-2 or IL-15, which must otherwise be provided in recombinant form during ex vivo culture or produced in sufficient amounts by surrounding immune cells in vivo [50]. Our data show that this advantage is retained upon the co-expression of the IL-15 superagonist RD-IL15, which in contrast to wildtype IL-15, contains part of IL-15Rα and carries the IL-15_N72D_ mutation that enhances the affinity for the IL-2/IL-15 receptor complex [32].

Unlike NK-92/hu14.18.28.z cells only expressing the GD_2_-CAR, NK-92/hu14.18.28.z_RD-IL15 cells remained viable to a high extent, continued to proliferate, and retained effective natural and CAR-mediated cytotoxicity in the absence of recombinant IL-2, indistinguishable from CAR-NK cells in the presence of IL-2. This autocrine activation of the IL-2R/IL-15R complex in RD-IL15-expressing cells was confirmed by continued phosphorylation of STAT5 as a downstream signaling molecule irrespective of the presence of IL-2. Importantly, permanent stimulation by RD-IL15 did not result in an exhausted phenotype of the cells as seen with IL-15 in other studies [40,41]. Expression of RD-IL15 also provided a selective advantage to the producer cells, facilitating self-enrichment after lentiviral transduction in the culture merely by IL-2 withdrawal. Similar factor independence and self-enrichment was previously observed upon the expression of wildtype IL-15 in NK-92 cells [30]. However, in this earlier study the cytokine was not secreted in significant amounts, whereas, here, RD-IL15 was found both bound to the surface of the NK-92/hu14.18.28.z_RD-IL15 producer cells and secreted into the culture medium at concentrations suitable to modulate the activity of bystander lymphocytes co-cultured with the CAR-NK cells.

IL-15 overlaps structurally and functionally to a large extent with IL-2, including the ability to promote the proliferation of activated T cells and the survival and functionality of NK cells. Nevertheless, there are also significant differences between these two cytokines, making IL-15 more preferable for cancer immunotherapy [51]. While IL-2 can promote activation-induced cell death of T cells, this is inhibited by IL-15. Furthermore, in contrast to IL-2, IL-15 has only a marginal effect on the activation of regulatory T cells. IL-15 also supports the maintenance of CD8^+^ memory T cells [52]. Due to its short half-life, in vivo efficacy of recombinant IL-15 is limited, with its stability depending on the availability of the receptor subunit IL-15Rα [53]. Therefore, IL-15/IL-15Rα fusion proteins similar to the RD-IL15 molecule used in our study have been developed as IL-15 superagonists, which can bind to the intermediate-affinity receptor complex of IL-2R/IL-15R β- and γ-chains in the absence of cross-presentation by IL-15Rα on neighboring cells and exhibit increased stability and in vivo efficacy.

Due to its particular importance for NK and CD8^+^ T cells [52], we focused in our co-culture assays on the influence of soluble factors from RD-IL15-expressing CAR-NK cells on donor-derived peripheral blood NK cells and cytotoxic T lymphocytes. Thereby, secreted factors from NK-92/hu14.18.28.z_RD-IL15 but not NK-92/hu14.18.28.z cells enhanced the proliferation of pNK cells from different donors in transwell cultures, which was more pronounced for cells with high CD56 levels. While we did not simultaneously analyze CD16 expression, these CD56^high^ cells may indeed represent the less differentiated CD56^bright^ NK cell subpopulation. In addition, exposure to soluble factors from RD-IL15-producing CAR-NK cells markedly increased natural cytotoxicity of innate lymphocytes against K562 erythroleukemia cells, which was much less pronounced upon co-culture with RD-IL15-negative NK-92/hu14.18.28.z cells, attributing this effect predominantly to the IL-15 superagonist. Transwell co-culture with NK-92/hu14.18.28.z_RD-IL15 cells also induced proliferation of CD8^+^ T cells to a similar extent as direct contact of the CTLs with allogeneic stimulator cells, while CAR-NK cells lacking the IL-15 superagonist were unable to induce this effect. Nevertheless, in this assay, stimulation by secreted RD-IL15 was less pronounced than that of recombinant IL-15 added to the culture in the absence of CAR-NK cells, which may have been due to partial consumption of the IL-15 superagonist and cytokines endogenously produced by activated T cells by the co-cultured CAR-NK cells, limiting availability to CD8^+^ T cells to a level insufficient for full activation.

Enhanced activity of endogenous NK cells and CTLs in the tumor microenvironment mediated by RD-IL15 can be expected to complement direct cytotoxicity of IL-15-producing CAR-NK cells and enhance overall antitumor efficacy. Nevertheless, also potential intensification of unwanted effects has to be considered. In a recent study in AML patients, the combination of systemic IL-15 superagonist N-803 with adoptive transfer of allogeneic NK cells exacerbated T cell-mediated rejection of the donor cells [54]. In the case of NK-92, treatment with a single dose of a CAR-engineered variant targeting ErbB2 (HER2) did not induce an immune response against the effector cells in our ongoing clinical trial in glioblastoma patients [28]. Nevertheless, this may be different upon repeated administration of CAR-NK cells, and it remains to be determined in future studies whether the beneficial effects of locally secreted IL-15 superagonist would come at the price of more limited persistence of allogeneic CAR effector cells in such a setting.

## 5. Conclusions

Taken together, the results from this exploratory in vitro study demonstrate high and selective antitumor activity of clinically relevant NK-92 cells engineered with a second-generation CAR targeting the disialoganglioside GD_2_. Constitutive expression of an IL-15 superagonist in these cells resulted in autocrine stimulation and independence from exogenous cytokine support. A similar approach may also be followed with donor-derived primary NK cells, which, in contrast to NK-92, do not require irradiation as a safety measure before infusion into patients, likely allowing longer-term engraftment and bypassing the need for repeated treatments. Importantly, secreted RD-IL15 also enhanced proliferation and activity of innate lymphocytes and cytotoxic T cells neighboring the CAR effectors, which may be relevant in a clinical setting for a concerted and effective antitumor attack by adoptively transferred and endogenous immune cells. Preparations are underway to now investigate the potential cooperation of RD-IL15-secreting CAR-NK cells and bystander immune cells in the tumor microenvironment in vivo in respective mouse tumor models.

## Data Availability

Data supporting the findings of this study are available within the article, the Supplementary Materials, or from the corresponding author upon reasonable request.

## Supplementary Materials

The following supporting information can be downloaded at: https://www.mdpi.com/article/10.3390/cancers15174310/s1, including in a single file: Figure S1: Binding of ganglidiomab-derived scFv-Fc protein to NK-92/hu14.18.28.z and NK-92/hu14.18.28.z_RD-IL15 cells; Figure S2: CAR-mediated lysis of tumor cells expressing a ganglidiomab scFv antibody domain as a GD_2_ surrogate; Figure S3: Analysis of a cell surface bound and secreted IL-15 superagonist; Figure S4: Effect of repeated stimulation on CAR-NK cells. Figure S5: Uncropped immunoblots of Figure 1C; Figure S6: Uncropped immunoblots of Figure 4A.
