# Supplementary material for: Co-Expression of an IL-15 Superagonist Facilitates Self-Enrichment of GD2-Targeted CAR-NK Cells and Mediates Potent Cell Killing in the Absence of IL-2
Source: Cancers (Basel). 2023 Aug 29;15(17):4310. doi: 10.3390/cancers15174310 (PMC10486391; doi:10.3390/cancers15174310)
Supplement: Supplementary file 1 [file cancers-15-04310-s001.zip › cancers-2497907-supplementary/cancers-2497907-supplementary.pdf]

Supplementary Materials

Co-Expression of an IL-15 Superagonist Facilitates Self-Enrichment of GD_2-Targeted CAR-NK Cells and Mediates Potent Cell Killing in the Absence of IL-2

Malena Bodden, Aline Häcker, Jasmin Röder, Anne Kiefer, Congcong Zhang, Anita Bhatti, Jordi Pfeifer Serrahima, Evelyn Ullrich, Ines Kühnel and Winfried S. Wels

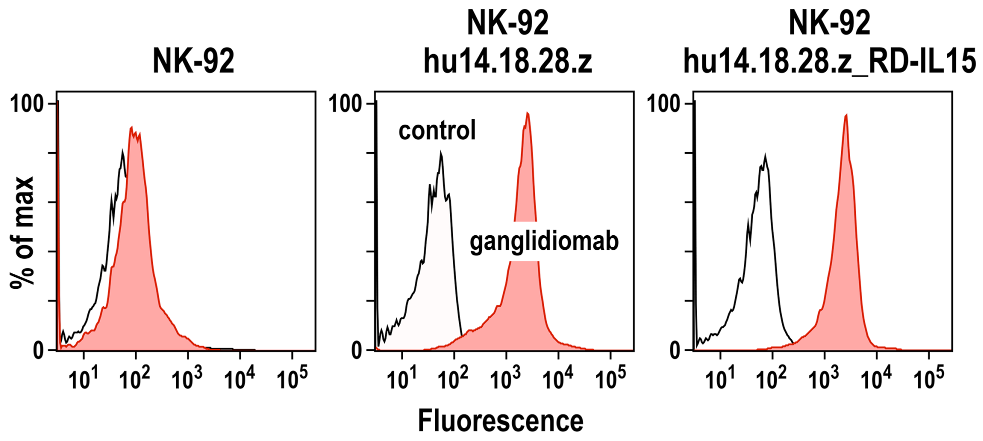

Figure S1. Binding of ganglidiomab-derived scFv-Fc protein to NK-92/hu14.18.28.z and NK-92/hu14.18.28.z_RD-IL15 cells. Binding of the recombinant anti-idiotypic antibody to the hu14.18 domain of the CAR was analyzed by flow cytometry using purified scFv-Fc protein and anti-human IgG secondary antibody (filled areas). Parental NK-92 cells and cells only stained with secondary antibody (black lines) were included as controls.

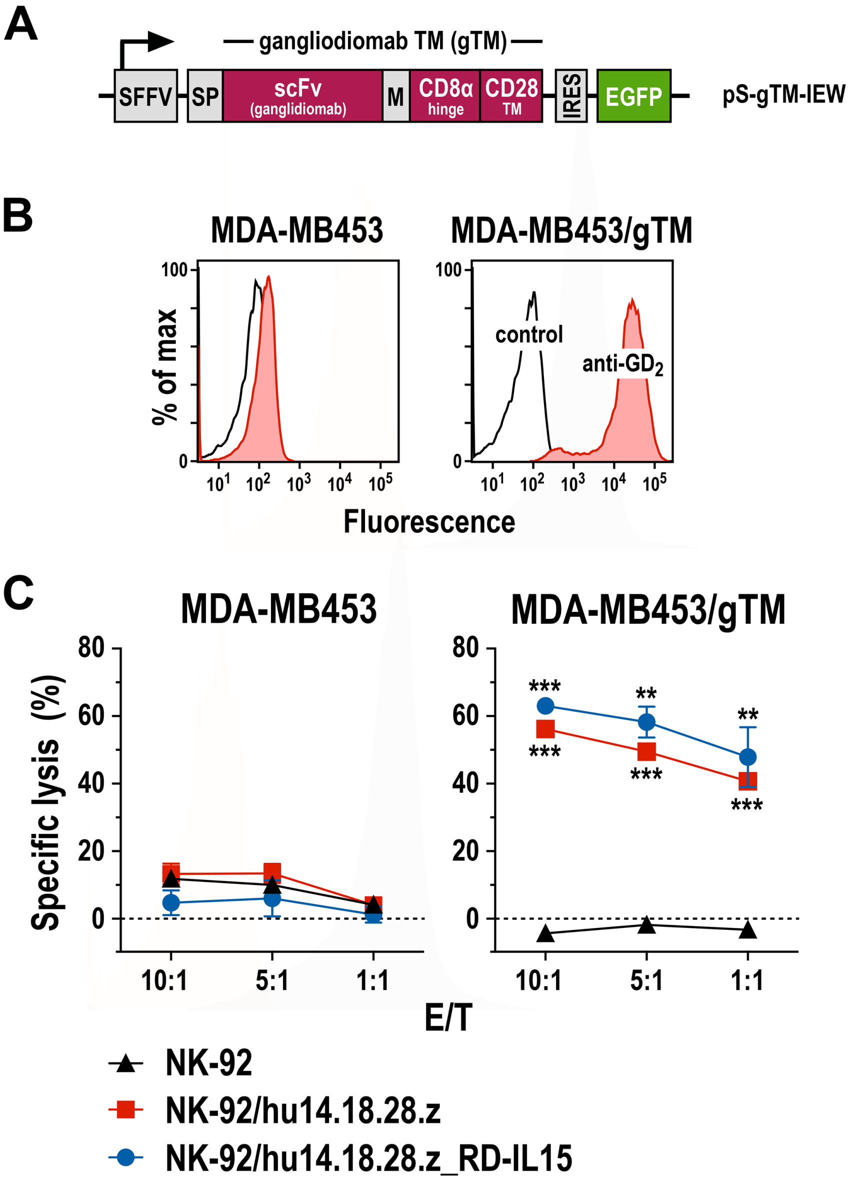

Figure S2. CAR-mediated lysis of tumor cells expressing a ganglidiomab scFv antibody domain as a GD_2 surrogate. (**A**) Lentiviral transfer plasmid encoding a membrane-anchored scFv fragment derived from anti-idiotypic antibody ganglidiomab (gTM) under the control of the Spleen Focus Forming Virus promoter (SFFV). The molecule consists of an immunoglobulin heavy chain signal peptide (SP), a ganglidiomab-derived scFv antibody fragment, a Myc-tag (M), a CD8α hinge region (CD8α) and the transmembrane domain of CD28. The transgene is followed by an internal ribosome entry site (IRES) and enhanced green fluorescent protein (EGFP) cDNA. (**B**) Surface expression of gTM on lentivirally transduced MDA-MB453/gTM cells was investigated by flow cytometry with anti-GD_2 antibody (filled areas). Unstained cells (black lines) and parental MDA-MB453 cells served as controls. (**C**) Cell killing activity of NK-92/hu14.18.28.z and NK-92/hu14.18.28.z_RD-IL15 cells against MDA-MB453/gTM and parental MDA-MB453 cells was investigated in flow cytometry-based cytotoxicity assays at the indicated effector to target (E/T) ratios after co-culture for 4 hours. Parental NK-92 cells were included for comparison. Mean values ± SD are shown; n=3; ***, $p < 0.001$; **, $p < 0.01$.

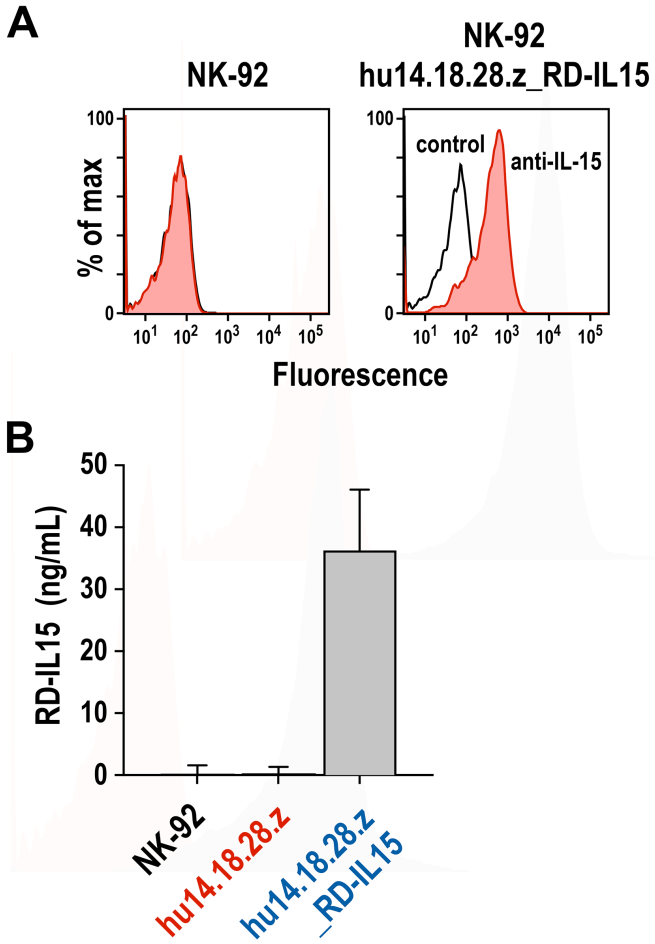

Figure S3. Secretion of IL-15 superagonist by NK-92/hu14.18.28.z_RD-IL15 cells. (**A**) RD-IL15 bound to the surface of NK-92/hu14.18.28.z_RD-IL15 cells was analyzed by flow cytometry with IL-15-specific antibody and AF647-conjugated secondary antibody (filled areas). Parental NK-92 cells and cells only stained with secondary antibody (black lines) served as controls. (**B**) RD-IL15 secreted into the culture supernatant by NK-92/hu14.18.28.z_RD-IL15 cells during a cultivation period of 3 days was measured by sandwich ELISA using an IL-15-specific capture antibody, and biotin-conjugated IL-15Rα-specific antibody and HRP-coupled streptavidin for detection. Recombinant His-tagged RD-IL15 served as a standard for quantification. Culture supernatants from parental NK-92 and NK-92/hu14.18.28.z cells served as controls. Mean values ± SD are shown; n=3.

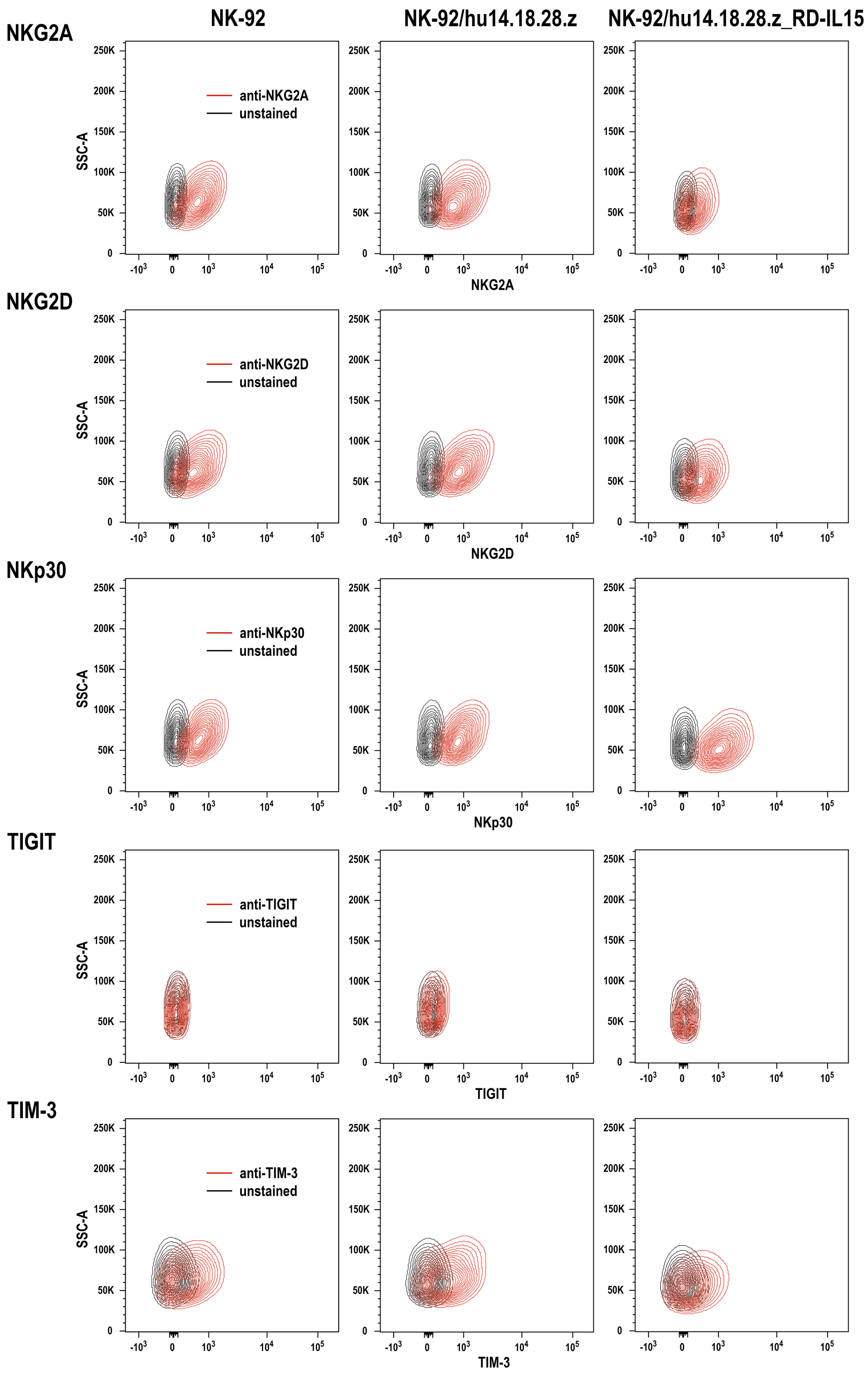
NK-92
NK-92/hu14.18.28.z
NK-92/hu14.18.28.z_RD-IL15
NKG2A
NKG2D
NKp30
TIGIT
TIM-3
anti-NKG2A
anti-NKG2D
anti-NKp30
anti-TIGIT
anti-TIM-3
unstained
SSC-A
250K
200K
150K
100K
50K
0
-10³
10³
10⁴
10⁵

Figure S4. Effect of repeated stimulation on CAR-NK cells. Parental NK-92, NK-92/hu14.18.28.z and NK-92/hu14.18.28.z_RD-IL15 cells were co-incubated with GD_2-positive EL4 cells at an E/T ratio of 1:2, with fresh target cells added after 24, 48 and 72 hours. One day later, cell surface expression of the indicated activating and inhibitory receptors and ligands was analyzed by flow cytometry. Representative contour plots of the data displayed in Figure 6 are shown.
