# Supplementary figures and images for: Co-Expression of an IL-15 Superagonist Facilitates Self-Enrichment of GD2-Targeted CAR-NK Cells and Mediates Potent Cell Killing in the Absence of IL-2
Source: Cancers (Basel). 2023 Aug 29;15(17):4310. doi: 10.3390/cancers15174310 (PMC10486391; doi:10.3390/cancers15174310)

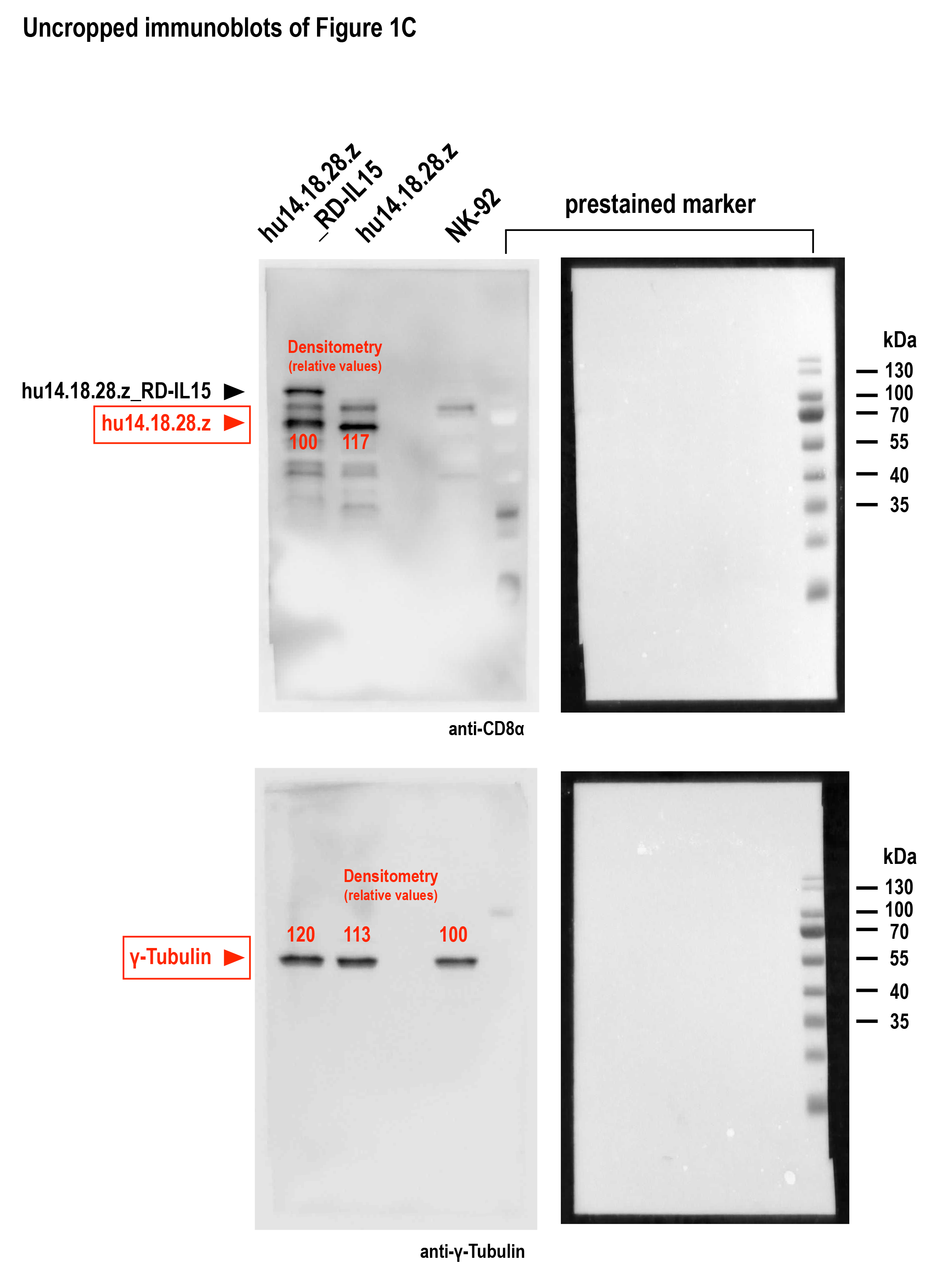

Supplement: Supplementary file 1 [file cancers-15-04310-s001.zip › cancers-2497907-supplementary/Figure S5.tif]

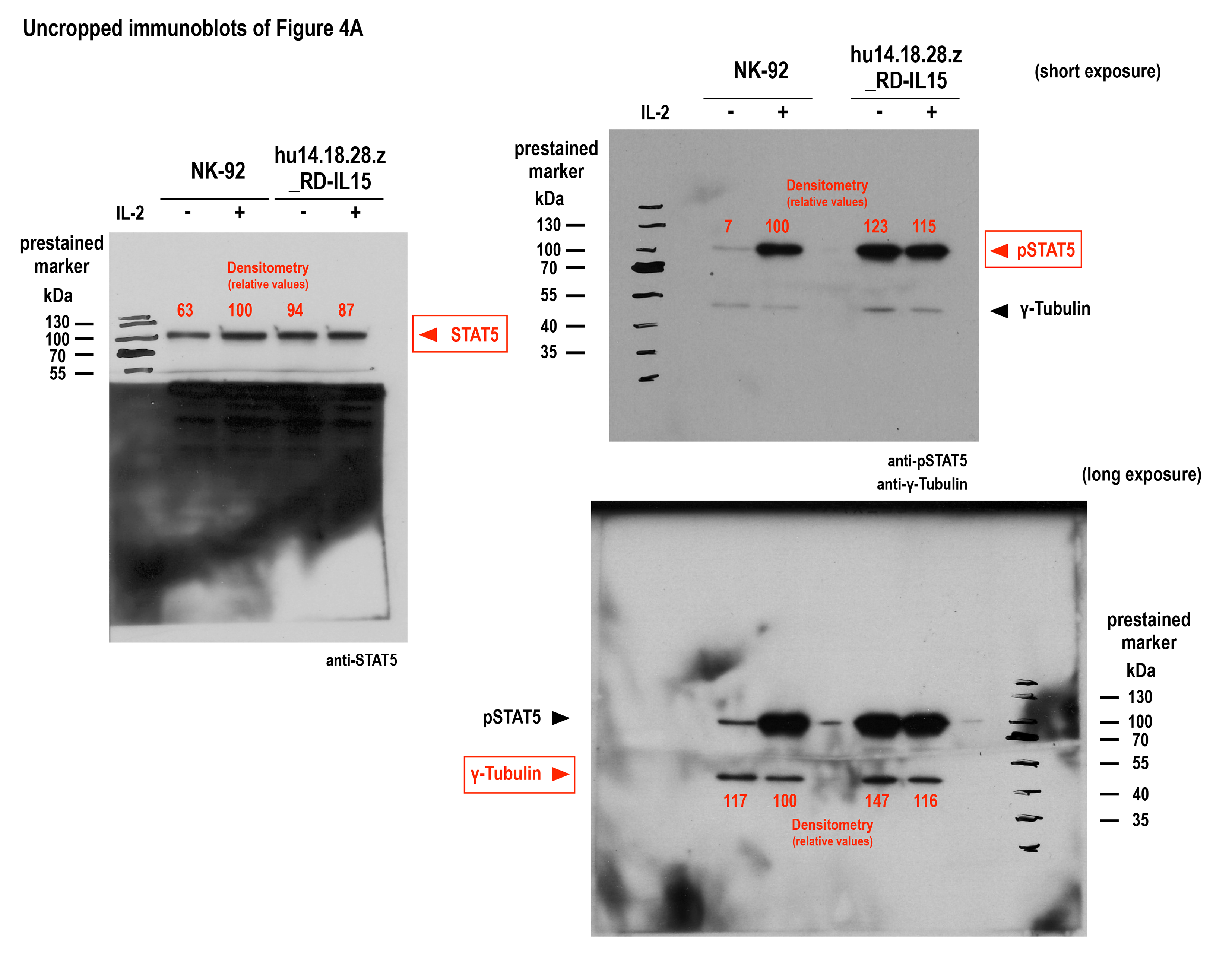

Supplement: Supplementary file 1 [file cancers-15-04310-s001.zip › cancers-2497907-supplementary/Figure S6.tif]
